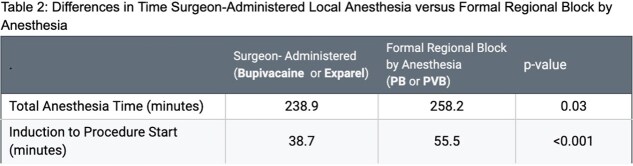# Comparing the Efficacy of Preoperative Regional Nerve Block Versus Intraoperatively Placed Exparel Block in Adolescents Undergoing Breast Reduction Surgery

**DOI:** 10.1093/asjof/ojaf018.016

**Published:** 2025-05-13

**Authors:** Cedar Slovacek, Madison Yeager, Caroline Kreh, Kevin Chen, Christina Plikaitis

**Affiliations:** Saint Louis University, St. Louis, MO; Saint Louis University, St. Louis, MO; Saint Louis University School of Medicine, St. Louis, MO; Saint Louis University, St. Louis, MO; Saint Louis University, St. Louis, MO

## Abstract

**Goals/Purpose:**

Breast reduction for macromastia is an effective and increasingly popular surgery in the United States. Since 2019, a 64% increase in cases has been seen, with over 80,000 cases performed annually. The psychosocial and physical benefits of breast reduction are well-documented in both the adult and adolescent populations. A variety of techniques aimed to improve postoperative pain control and recovery for adult patients have been described in the literature; however, each technique not only has varying effectiveness, but is also associated with differences in additional operative time, cost, risks, and availability. Moreover, this data has not been well-described in the adolescent population.

Our institution follows a large population of adolescent patients undergoing breast reduction by a single surgeon. Over the last twelve years, we have evolved in our perioperative pain control strategies from initial simple peri-incisional infiltration of local anesthetic, to formal preoperative regional nerve blocks performed by the anesthesia team, to, most recently, use of intraoperative Exparel for PECS 1 and 2 nerve blocks placed by the surgical team.

In this study, we sought to determine the effect of each of these three techniques on reported early post-operative pain levels, narcotic requirements, and incidence of postoperative nausea and vomiting in adolescent patients undergoing elective breast surgery.

**Methods/Technique:**

We conducted a retrospective review of all adolescent patients who underwent bilateral breast reduction from 2012-2024 at a single institution by a single surgeon. Patients were excluded if they underwent unilateral reduction, did not receive any of the above perioperative pain control techniques, or were >20 years old at time of surgery. Patients were categorized by the type of local or regional anesthetic blocks they received. This included 1) intraoperative peri-incisional bupivacaine, regional block by the anesthesia team, either 2) paravertebral (PVB) or 3) pectoral (PB), or 4) intraoperative liposomal bupivacaine (Exparel), which was placed by the surgeon as bilateral PECS 1 and 2 blocks.

Chart review was performed to collect patient demographics, length of surgery and anesthesia time, pain medication required during postoperative hospital stay (calculated in oral morphine equivalents (MEQ)), postoperative pain scores, episodes of emesis, and complications associated with anesthetic technique were recorded for each group. Statistical analysis was performed using one way ANOVA testing and post hoc analysis, where appropriate.

**Results/Complications:**

88 patients met inclusion criteria. 17% (N=15) received intraoperative peri-incisional bupivacaine, 8% (N=7) received PB, 28.4% (N=25) received PVB, and 46.6% (N=41) received liposomal bupivacaine. There were no significant differences between groups in terms of BMI, age, length of procedure, or total specimen weight (Table 1).

One way ANOVA test demonstrated significant differences in post-operative MEQ required between groups (p <0.001). Specifically, post hoc analysis revealed that the Exparel group required significantly less narcotic pain medication (MEQ) during their hospital stay than those that received bupivacaine, PB, or PVB (p<0.001, p = 0.035, p = 0.012). Additionally, patients that underwent regional blocks (ie: PB or PVB) were under anesthesia for significantly longer time than patients who received surgeon-administered anesthetic (ie: bupivacaine or Exparel) (258 vs 239 minutes, p = 0.03). When performed preoperatively, regional blocks also increased the time from induction to procedure start time (38.7 vs 55.5 minutes, p < 0.001) (Table 2).

There were no differences in episodes of emesis (p = 0.20), or average postoperative pain scores (p= 0.93). In regard to complications, one patient who underwent PVB with anesthesia suffered postoperative unilateral lower extremity paresthesias lasting 3 months, whereas there were no complications among the patients that received PB or surgeon-adminstered bupivacaine or Exparel.

**Conclusion:**

Among adolescents undergoing elective breast reduction surgery, intraoperative placement of Exparel PECS 1 and 2 blocks was associated with significantly less postoperative narcotic use than peri-incisional bupivacaine or a formal regional block performed by anesthesia. Additionally, both intraoperative surgeon-performed techniques were associated with significantly shorter time under anesthesia and time from induction to procedure start when compared against regional blocks. Our data suggests that intraoperative placement of Exparel PECS 1 and 2 blocks is effective for pain control and can minimize narcotic use in this vulnerable population of adolescent patients undergoing breast reduction while avoiding the increased time under anesthesia and potential risks of formal regional blocks. We believe this technique is a safe and effective alternative to formal regional blocks for augmenting perioperative pain management while also decreasing narcotic use in adolescent patients undergoing breast reduction.

**Figure:**
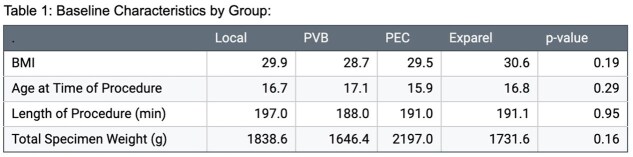

**Figure:**